# Are We Making Progress? Assessing Goal-Directed Behaviors in Leadership Development Programs

**DOI:** 10.3389/fpsyg.2019.01345

**Published:** 2019-06-11

**Authors:** Ferran Velasco, Joan Manuel Batista-Foguet, Robert J. Emmerling

**Affiliations:** People Management and Organisation, ESADE Business School, Universitat Ramon Llull, Barcelona, Spain

**Keywords:** goal-directed behaviors, goal setting, goal striving, leadership development, scale development

## Abstract

Leadership development programs increasingly help participants engage in their career transitions. Therefore, these programs lead participants to establish not only development goals, which usually involve the improvement of a specific leadership competency, but also goals that relate to career advancement or to achieving a more general life aspiration. Assessing goal attainment, as a measure of program impact, may take years as goals vary greatly in terms of nature, timeframe, and domain. The purpose of this study was to overcome this challenge by providing a measure of goal progress as a necessary antecedent of goal attainment, and which we operationalize through a general scale of goal-directed behaviors. Subject-matter experts assessed the content validity of the measure. Factor analysis, using three samples, revealed four dimensions identified as Sharing Information, Seeking Information, Revising the Plan, and Enacting the Plan. This new scale allows data collection as early as a few months after setting the goals, which can provide practitioners with an earlier indication of program impact and facilitate future academic studies in this field.

## Introduction

Leadership development programs aim to help participants in acquiring and developing the intrapersonal and interpersonal competencies that are necessary for leading teams and organizations more effectively (Day, 2000; Day et al., 2014), such as emotional awareness, adaptability, empathy, and conflict management. In executive education, these programs often use 360-feedback tools to provide an assessment of these competencies, which participants then use as a reference to set their improvement goals and define their leadership development^1^ plans (Brett and Atwater, 2001).

Business schools, however, increasingly recognize the fact that many professionals join these programs to embark on a personal or professional transition (Kets de Vries and Korotov, 2007) and in response, they have started promoting such future career or personal transitions as part of their leadership development programs (Russon and Reinelt, 2004). Consequently, improvement plans that participants write often combine short-term goals related to leadership competencies (e.g., *to improve my communication skills*) with longer term and more aspirational career or personal goals (e.g., *to become a general manager*).

Since these programs are costly and demand substantial personal effort, stakeholders expect them to be effective and to help participants accomplish their goals. Goal attainment, i.e., the degree to which a participant achieves the set goals, is thus considered a key outcome, and its measurement is fundamental to establishing program success (Toegel and Conger, 2003). However, assessing the impact that such training interventions has on individual change constitutes a challenge, as change is “an individualized and serendipitous experience” (Bernthal et al., 2001, p. 507), and its study is inherently longitudinal. It is only natural that leaders, after completing their program, gradually disengage from the university, business school, or organization that imparted the course, and as a result, data collection becomes more challenging as time goes by. This may explain why it is scarcely known whether leaders actually make progress toward their goals.

The need for measurement scales assessing the short-term impact of these programs on individual change has been acknowledged in the leadership development literature (Hooijberg and Lane, 2009). With this study, we respond to this need by providing a general scale of goal-directed behaviors (GDB) that measures *goal progress*, a necessary antecedent of goal attainment. Unlike previous goal-specific constructs, this new construct reflects the level of engagement in goal pursuit in general and can therefore be applied regardless of the number and nature of the goals.

Instruments for measuring goal attainment found in the leadership development literature have two important limitations which our scale overcomes: first, not being general enough to cover multiple goal domains and second, having to wait too long for data collection. For example, a common option used to measure goal attainment is to administer a second 360-feedback survey at a later date. The survey, however, would only apply to a fraction of the goals (to those concerning competencies as assessed by the 360-feedback survey, but not to those related to the job, career, or personal domains). Additionally, it can take more than a year for the effects of training to start being visible to others (Cherniss et al., 1998). By then, leadership programs have long been completed, thus making a second 360-feedback to assess goal progress a challenge to implement. Black and Earnest (2009), also recognizing the need in the literature for an instrument that evaluates the impact of such training programs, developed a self-reported scale which assesses the improvement of specific skills. While this scale makes data collection easier, it also applies to only a fraction of the goals, i.e., those related to specific competencies as evaluated by the scale. Acknowledging the need for a more general measure of goal attainment that can be applicable to multiple heterogeneous goals, Spence (2007) developed the Goal Attainment Scale (GAS), which is a weighted average score of the degree of success of all goals, with each goal being weighted by a perceived difficulty rating. This measure was developed for long-term coaching interventions, in which the coach guides the client along the goal-striving process. However, in the context of leadership development programs, the goal-striving process can easily take years and therefore such costly coaching interventions are seldom offered.

Measuring goal-directed behaviors as an early indicator of goal attainment is not new in goal setting literature (e.g., Ajzen, 1991; Perugini and Bagozzi, 2001; Leone et al., 2004). However, these measures also share the limitation of being goal specific and therefore can only be applied in the domain of their study. For example, a measure of *time spent providing feedback to improve people’s performance* is usually specific to the goal of *improving your competency in developing others*, and therefore cannot be used to assess goal progress toward multiple goals pertaining to multiple domains.

Since our general scale of GDB overcomes the aforementioned limitations (i.e., restriction to a specific goal domain and difficulty in data collection), it can be used to assess progress toward multiple goals in multiple domains and it can be applied as early as a few months after goals are set, a timeframe that facilitates data collection as participants are likely to be (either physically or emotionally) still involved in the program.

The present article starts with the definition of the GDB construct. It then proceeds with an overview of goal setting theory (i.e., Locke and Latham, 1990, 2002; Gollwitzer, 1999; Latham, 2004; Seijits and Latham, 2005) as the framework leading to the different dimensions of the GDB construct, the hypothesis for the measurement model, and the justification of the variables in the nomological network that are later considered for construct validation. In section “Method,” we describe the steps followed to develop and validate the scale, a process that led to a final 18-item scale tapping four behaviors: Sharing Information, Seeking Information, Revising the Plan, and Enacting the Plan. The study is based on data from business executives taking part in a leadership development program designed around Intentional Change Theory (Boyatzis, 2006, 2008). We conclude by highlighting the theoretical contribution and the practical advantages of having a general scale of GDB that can be applied soon after the goals are set. Limitations of the study are discussed and directions for future research using the general scale of GDB are suggested.

## Indicators of Goal Attainment

Goal setting theory states that goals regulate human behavior by providing purpose or intent, and that there is a positive relationship between goal difficulty and task performance. This relationship is explained by four possible mechanisms: goals (1) divert the direction of action toward goal-related behaviors, (2) energize people, (3) increase people’s persistence in their striving toward achieving the goal, and (4) encourage people to discover task-specific knowledge and strategies on how tasks should be better performed (Locke and Latham, 1990, 2002; Latham, 2004). Goal setting theory therefore indicates that focusing on goal-directed behaviors (GDB) is one of the mechanisms that helps individuals to achieve their goals.

The study of goal-directed behaviors has accumulated more than 30 years of research. Academics have been mostly concerned with understanding the psychological mechanisms that explain the variance in goal-directed human behavior. Several theoretical models of GDB have been proposed and empirically validated in a variety of contexts. Each model aims at improving the explanatory power of GDB, a construct that has mostly been treated as the dependent variable of the models.

In the Theory of Reasoned Action (Fishbein and Ajzen, 1975), *intention* to perform the behavior was asserted to be the immediate antecedent of the behavior in question. This model was later refined by incorporating *perceived behavioral control* as another determinant of behavior (Ajzen, 1991) and this has become one of the most prominent models in the field of behavioral goals: the Theory of Planned Behavior (TPB).

The TPB model was further expanded and deepened introducing new constructs, *anticipated emotions* and *desire to perform the action*. Anticipated emotions are related to the predicted consequences of achieving the goal, emotions that trigger the desire and the subsequent intentions to act (Perugini and Bagozzi, 2001). The anticipated effects of goal attainment are therefore more thoroughly captured in this new model, which the authors named the model of Goal-Directed Behaviors (GDB).

Whereas studies based on the TPB usually measure behavior as the target of all the independent variables of the model (i.e., the behavior or task becomes the end goal in itself), studies based on the GDB model treat behaviors as a means to an end-state goal (e.g., *asking for feedback after a presentation* – the GDB – *in order to improve my communication skills* – the end goal). Since engaging in GDB to achieve an ultimate goal is what managers in leadership development programs typically do, we might ask whether it is therefore possible to apply any of the scales used in the GDB models to our domain of interest.

Evidence for the validity of such models emanates from context-specific studies which are not closely related to leadership development. In such studies, the nature of the GDB and that of the end goal itself are perfectly determined, and as a result, constructs are measured by context-specific scales. A typical example is “I intend to study handbooks to learn how to use the statistical package during the next 4 weeks,” a measure that is specific to the goal of getting a good examination score (Leone et al., 2004, p. 1956).

Existing context-specific GDB scales are unfortunately not applicable in the domain of leadership development programs, where different individuals can set different numbers of goals and goals of a different nature. Therefore, to measure goal progress as an assessment of the short-term impact that these programs have on individual change, we need a new (and general) scale of GDB that is applicable in this domain.

## Defining Goal-Directed Behaviors

As a preliminary step in scale development, it is necessary to have a proper definition of the construct that suits the domain of interest (Hinkin, 1995), for which we require an understanding of the nature of GDB in the context of leadership development programs.

Managers who participate in leadership development programs usually have a great deal of discretion in writing out their goals and action plans. This is even more so when these programs are part of executive education courses in business school settings, as participants are not likely to have program constraints coming from their work organizations. As previously mentioned, goals and development plans typically relate to the improvement of a specific skill or competency, but may also relate to career advancement or even to the achievement of a more general life aspiration.

A disparity of goals is likely to generate a disparity of action plans, and hence a multiplicity of intentions to put a wide variety of behaviors into practice. Even participants who set one single goal may plan multiple actions or behaviors, all aimed at achieving the goal. An analysis done in a recent study that comprised 189 goals and 1,028 action plans written out by executives from a leading business school in Europe (the context of our study) provides compelling evidence of this assertion^2^. One participant set the goal *to improve my communication skills*. She then specified 10 actions, which included *to record myself in a presentation to analyze my weaknesses*, *to do a Coursera course in public speaking*, and *to practice some of the competencies in front of my project group*. Each of these actions involved the display of a different behavior or sets of behaviors, all of them directed to achieving the goal (to improve the communication skills).

Measuring GDB in leadership development programs therefore requires a general scale that can be used to measure behaviors independently of their nature and number. Consequently, the definition of the GDB construct that we propose is context-neutral, namely *the enactment of behaviors that facilitate goal attainment.*

## Dimensionality of Goal-Directed Behaviors in Leadership Development Programs

When developing a new scale, it must be ensured that items that measure the construct cover the theoretical domain of interest. Therefore, the first step is to establish and define the dimensions of the construct, dimensions that can be derived from theory (deductive approach), from observations (inductive approach), or from both (Hinkin, 1998).

An examination of the existing theory on goal setting and goal striving, and a systematic review of the literature on leadership development programs using multisource feedback^3^, allowed us to derive three dimensions of our GDB construct: *Sharing Information, Seeking Information*, and *Enacting the Plan*. Direct observations, which allowed us to assess face validity of these three theory-driven dimensions, uncovered a fourth one: *Revising the Plan.* Below, we discuss each of these four dimensions in detail. We then present a model of GDB by hypothesizing how the dimensions are related to each other.

### Sharing Information

Goal setting literature has shown that, for goals to be effective, there must be commitment to the goals (Locke and Latham, 1990). Goal commitment, defined as an individual’s determination to reach a goal (Locke et al., 1988), increases if the goals are made public. Research shows that sharing goal intentions and action plans with others increases goal commitment (Hollenbeck et al., 1989a,b; Epton et al., 2017). Therefore, those who share their goals and action plans with more people are likely to also strive with more determination toward achieving the goals. Many leadership development programs assess their participants’ managerial competencies using multisource feedback tools. Multisource feedback entails receiving feedback from multiple sources, usually direct reports, peers, co-workers, and managers (London and Smither, 1995). Research strongly suggests that when this feedback is discussed with the boss, the participants’ perceived accountability for the goals increases, and as a consequence, their performance improves (London et al., 1997; Toegel and Conger, 2003). We therefore conclude that sharing information with others about the goals, action plans, or the feedback received during the training program is a dimension that our GDB construct should measure. We define this dimension as *sharing information with others related to feedback details, goal intentions, or action plans.*

### Seeking Information

Challenging, specific goals encourage individuals to discover task-specific knowledge or strategies on how tasks can be better performed. This behavior is one of the mediating mechanisms that explain an increase in performance (Locke and Latham, 1990, 2002; Latham, 2004). When individuals do not have the ability to perform the task or the knowledge on how to best achieve their goals, then the acquisition of knowledge and skills, rather than the increase in effort and persistence, becomes a salient mechanism for goal achievement (Seijits and Latham, 2005). Research also reveals that discussing and clarifying multisource feedback with raters, or discussing goals or action plans with others, has a positive effect on rating improvement over time (Toegel and Conger, 2003; Smither et al., 2004), and exerts a positive influence on goal attainment (Hazucha, 1993; Smither et al., 2004).

Goal setting is also more effective when feedback about the progress toward the goals becomes available to the individual during goal striving. Seeking information to monitor and evaluate progress toward goal attainment enhances metacognition and facilitates self-regulatory strategies to better achieve the goals (Locke and Latham, 1990). We therefore conclude that *Seeking Information,* whether as a cognitive strategy to learn how to better achieve the goals, or as a metacognitive strategy to obtain feedback on the progress toward the goals, is another relevant domain that our GDB construct should tap in the context of leadership development programs. We define this second dimension as *seeking information that could be useful in improving the action plan or the strategy to achieve the goals.*

### Enacting the Plan

Challenging, specific goals also direct actions toward goal-related behaviors, another of the mediating mechanisms that lead to higher performance (Locke and Latham, 1990, 2002; Latham, 2004). However, goal striving starts when the individual makes the transition from goal intentions to action. Goal intentions express *what* the individual intends to achieve. Once this decision is made, the mind-set changes into *how*: i.e., to determine the best course of action to be implemented in order to achieve the goal (Gollwitzer et al., 1990). Research in goal striving shows that action initiation is facilitated when individuals have clear mental anticipations of the behaviors most instrumental to meeting their goals (Gollwitzer, 1999). These mental anticipations or plans that specify the *how*, *where*, *when*, or *with whom* the goal is to be achieved are referred to as implementation intentions. A meta-analysis by Gollwitzer and Sheeran (2006) provides compelling evidence of the positive effects that forming implementation intentions has on triggering action and on goal achievement.

Once goals are set after completing a leadership development program, managers typically form their implementation intentions by means of an action plan. Such plans therefore reflect their mental anticipation of how to best achieve their set goals. Not surprisingly, several academic studies use the degree of plan implementation as a measure of progress, and as an early outcome of program success (Toegel and Conger, 2003; Hooijberg and Lane, 2009). We therefore claim that our GDB construct should also tap *Enacting the Plan*, a dimension we define as *enacting the action plan and making progress toward achieving the goals*.

To guard against the theoretically derived dimensions not covering all the domains of GDB, we then explored potential additional dimensions of the construct using the inductive approach, which involves the analysis of firsthand account of GDB (Hinkin, 1995). To obtain a purposeful sample of individuals who highly engaged in GDB, candidates for the interviews were selected on the basis of their past participation in the same or similar leadership development programs, and on their assent to having achieved their goals. We used semi-structured interviews to guide participants in their account of the specific behaviors, steps, or actions that they had engaged in since setting their personal goals and writing their corresponding action plans. Interviews were conducted and transcribed verbatim by the first researcher, who then coded actions according to the three theory-driven behaviors. We stopped after 10 interviews since concept saturation was reached after a few interviews. Data analysis led to the emergence of an additional dimension, Revising the Plan.

### Revising the Plan

Most behaviors observed in the interviews could be clearly classified under one of the three theoretically derived dimensions. However, a fourth domain emerged: some behaviors were related to the adaptation of plans to better achieve the goals, e.g., *After speaking with some experts I changed my plan and targeted a different set of multinational companies for job interviews*. Demonstrating flexibility to change the plans or adapt the strategy to attain the goals was a recurrent behavior observed in the interviews. Consequently, *Revising the Plan* was added as a fourth dimension of GDB, a dimension that we defined as *changing or adapting the action plan to attain the goals.*

## A Model of Goal-Directed Behaviors in Leadership Development Programs

It follows from the above that GDB is an aggregate construct (Law et al., 1998) as it is formed as a combination of four dimensions, which we hypothesized not to be independent of one another.

Since sharing goal intentions with others is likely to increase commitment toward the goals (Hollenbeck et al., 1989a,b; Epton et al., 2017) and to positively influence goal achievement (Hazucha, 1993; Antonioni, 1996; Smither et al., 2004), we expected that the more people with whom participants share their goals and plans, the more likely it is that they will engage in acquiring additional information and searching strategies to attain the goals, and in putting some of the actions into practice. We therefore hypothesized:

H1: Sharing Information is positively associated with Seeking Information.

H2: Sharing Information is positively associated with Enacting the Plan.

When goals are complex or challenging, as is often the case in leadership development programs, searching for information or for new strategies on how to progress toward the goals is a key mechanism for goal attainment (Locke and Latham, 1990, 2002). The information acquired, whether it comes from discussing feedback with others (Toegel and Conger, 2003; Smither et al., 2004), from reviewing plans and progress with others (Hazucha, 1993), or simply from inquiring through other external sources (e.g., through internet or through attending a seminar), is likely to help participants design a more effective action plan. Moreover, individuals who engage in seeking information with the aim of better attaining the goals are likely to feel more encouraged to put the action plans into practice. We therefore hypothesize that:

H3: Seeking Information is positively associated with Enacting the Plan.

Finally, self-regulatory strategies, such as seeking information or feedback to evaluate progress toward the goals, are likely to promote changes in behaviors and in the course of action to better attain the goals (Slocum et al., 2002; Harkin et al., 2016). Since information and feedback are likely to make discrepancies between the present state and the desired end goal more salient, individuals are likely to think of ways of adapting the present course of action to better attain the goals. We therefore hypothesized that people who engage in seeking information to assess the adequacy of their action plan, or their progress toward the goals, are more likely to revise their action plans to make them more effective. In turn, revised and better plans are more likely to encourage and facilitate enacting the plan. Hence:

H4: Seeking Information is positively associated with Revising the Plan.

*H5: Revising the Plan is positively associated with Enacting the Plan*.

**Figure 1:**
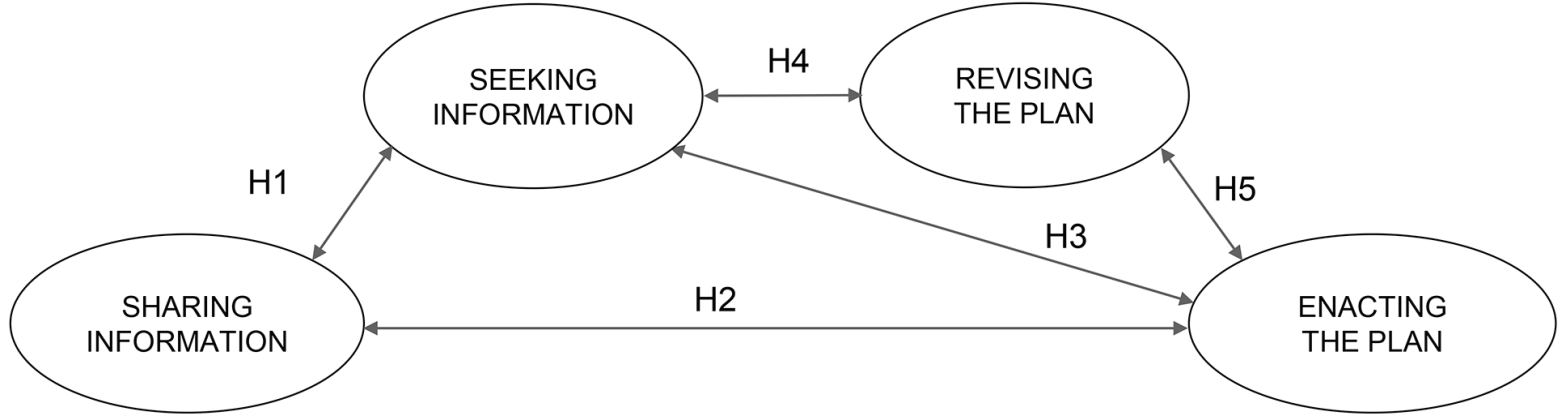
Model of GDB.

Taken together, our hypothesized relationships among the four dimensions lead to our proposed model of GDB, as illustrated in Figure 1.

## Conceptual Relationships with Other Related Constructs

When developing a new scale, it is also important to establish the conceptual relationships between the newly developed scale and related constructs in the domain of the study, which in our case is goal attainment. Each of the related constructs presented below has been well validated and the scales of all of them have been broadly tested in the literature, thus constituting a good nomological network for validating our new GDB scale.

### Goal Commitment

Since goals vary a lot from individual to individual, we used Klein et al.’s (2014), p. 222 target-free measure of goal commitment, which they conceptualize as “a volitional psychological bond reflecting dedication to and responsibility for a particular target.” Goal commitment is recognized as an essential moderator between goal level and performance (Locke and Latham, 1990; Latham, 2004; Latham and Locke, 2007), and there is extensive evidence of its significant effect on performance and goal achievement (e.g., Wofford et al., 1992; Slocum et al., 2002). Goal commitment shields goal pursuit (Shah et al., 2002) and encourages individuals to enact behaviors or actions directed to achieve the goals (Slocum et al., 2002). Goal commitment has also been found to be positively related to the discovery of strategies to attain the goal (Early et al., 1992), which is likely to lead to information-seeking behaviors. Research also shows that when goals are made public, i.e., when individuals share their goals and action plans with others, goal commitment increases (Hollenbeck et al., 1989a,b). In view of the above, we expected goal commitment to be positively correlated to *Enacting the Plan, Seeking Information,* and *Sharing Information.*

### Learning Goal Orientation

Learning goal orientation (LGO) measures the disposition toward developing ability in achievement situations (VandeWalle, 1997). Individuals with a high LGO are more open to new experiences (Payne et al., 2007) and tend to interpret feedback as useful for correcting errors and improving competencies. Consequently, these individuals are more likely to use effective learning strategies (Locke et al., 1981; Wood et al., 2013), and to share information as a means to actively engage in feedback-seeking behaviors (VandeWalle et al., 2001; Payne et al., 2007). LGO has also been shown to be positively associated with the achievement of performance goals (Latham and Locke, 2007; Taing et al., 2013), and consequently with the enactment of behaviors and actions planned for that purpose. In view of the above, we argued that LGO should also show positive correlations with our new measure of GDB, specifically with *Sharing Information, Seeking Information,* and *Enacting the Plan.*

### Avoiding Performance Goal Orientation

Avoiding performance goal orientation (APGO) measures the tendency to avoid exposing one’s lack of ability and to avoid negative judgment from others (VandeWalle, 1997). Individuals with a high APGO tend to interpret feedback as evaluative and judgmental, and are therefore less likely to see its usefulness for engaging in developing competencies needed to achieve their goals (VandeWalle et al., 2001). Research shows APGO to be negatively correlated with feedback seeking (Payne et al., 2007) and with job and performance outcomes (VandeWalle et al., 2001). Consequently, we expected APGO to be negatively associated with *Seeking Information* and *Enacting the Plan.*

### Self-Efficacy

Self-efficacy measures people’s beliefs in their capabilities to perform the behaviors needed to achieve their goals (Bandura, 2013). We chose Chen et al. (2001) general self-efficacy scale, as it is applicable to any situation, and is thus more appropriate for the context of leadership development programs, where individuals can set goals in a wide range of domains. Individuals with a high general self-efficacy are more likely to engage in effective knowledge acquisition and strategy development activities in the pursuit of achieving goals (Bandura, 2013). People with high self-efficacy also tend to be more persistent in the face of difficulties, since they are convinced they can succeed. Research shows that self-efficacy has a positive effect on goal-directed behaviors (Slocum et al., 2002), the search for task-specific knowledge or strategies (Latham, 2004) (i.e., seeking information), and goal achievement (Locke and Latham, 1990; Latham and Locke, 2007). Hence, we hypothesized that self-efficacy would be positively correlated with our measure of GDB, especially with *Seeking Information* and *Enacting the Plan.*

### Proving Performance Goal Orientation

Proving performance goal orientation (PPGO) measures the tendency to set achievable goals that allow one to prove one’s ability to gain favorable judgment from others (VandeWalle, 1997). Unlike APGO or LGO, PPGO has been shown to be unrelated to effort and task performance (VandeWalle et al., 2001), and to feedback seeking (Payne et al., 2007). Consequently, we predicted that PPGO should be unrelated to our measure of GDB. For the purpose of our study, the three dispositions of goal orientation – LGO, APGO, and PPGO – were measured using VandeWalle’s (1997) three-dimensional scale.

### Empathic Concern

Empathic concern (EC) measures the tendency to experience “other-oriented feelings of sympathy and concern for unfortunate others” (Davis, 1983, p. 114). Neurological studies show that leaders who possess high levels of EC are more likely to engage in social–emotional relational tasks, which activates the default-mode network in the brain (Boyatzis et al., 2014). In contrast, goal setting activates a different and competing network called task-positive (Boyatzis et al., 2014). We therefore predicted a lack of association between EC and GDB or a mild negative one.

In the following section, we describe the steps taken to develop and validate our new self-reported general scale of GDB.

## Method and Results

**Figure 2:**
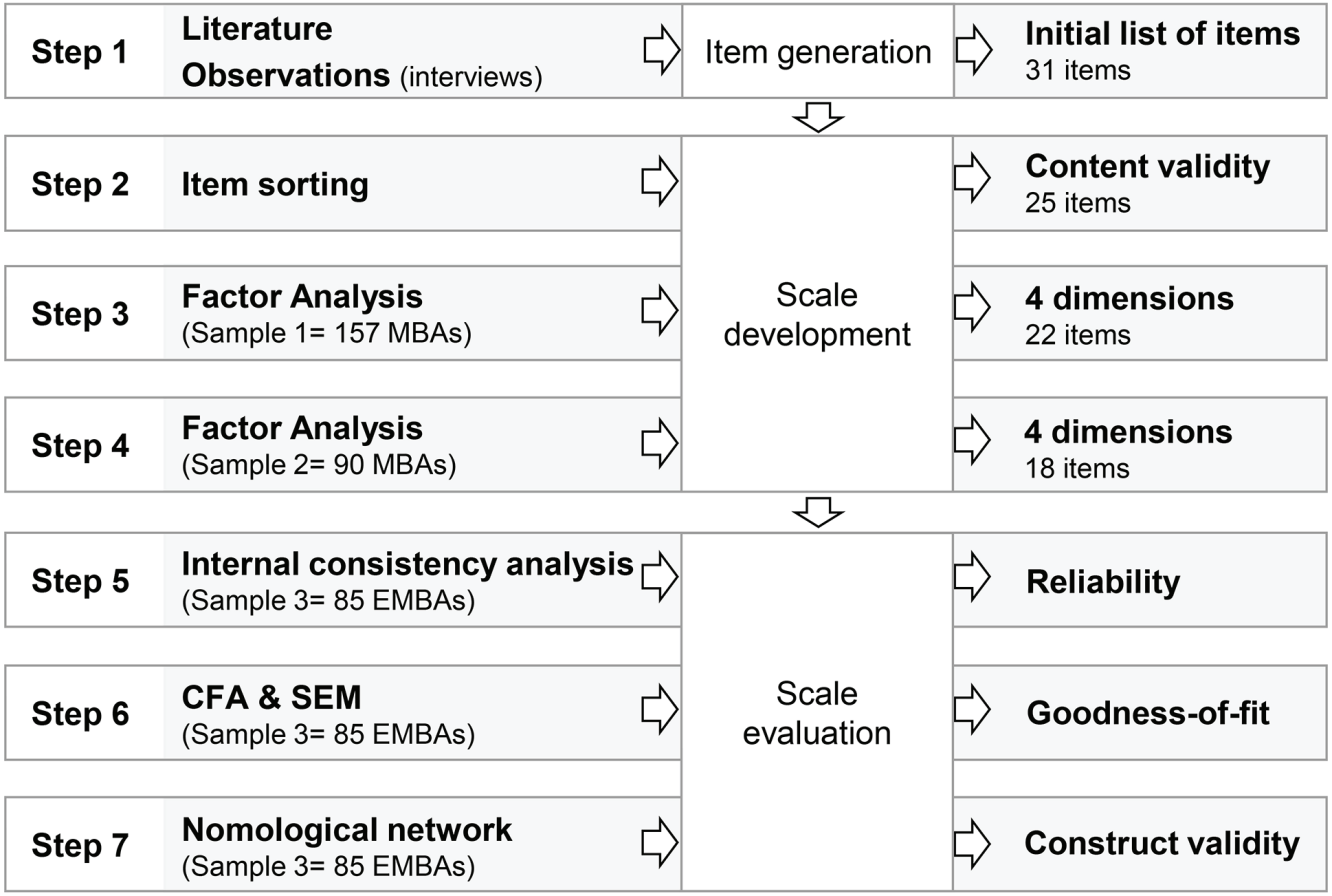
Stages followed to develop the general scale of GDB.

To develop and validate a general scale of GDB, we followed Hinkin’s (1995, 1998) framework for scale development, which is still considered to be a good standard for developing scales that aim at measuring behaviors in organizations (e.g., Djurdjevic et al., 2017). Figure 2 illustrates the three stages and the steps followed in this study. In the first stage (step 1), a pool of items was generated. In the second stage (steps 2, 3, and 4), the scale was developed through the rewording and elimination of items. In the third and final stage (steps 5, 6, and 7), the goodness-of-model fit was assessed, and the psychometric properties of the scale were evaluated.

The scale was developed and evaluated with data from participants of a leadership development program in a leading European business school. The program was designed around Intentional Change Theory (Boyatzis, 2006, 2008), which holds that personal change is more likely to occur if the change process is anchored in one’s vision, hopes, and aspirations (as opposed to just focusing on the weaknesses that need fixing). Participants were therefore encouraged to first describe their career and personal aspirations before receiving and interpreting their 360-feedback. As a result of this process, development plans tend to integrate a greater variety of goals and action plans (e.g., some related to the development of competencies and some more aspirational in nature). This vision-based approach makes participants more open to new ideas and experimentation (Boyatzis, 2008; Mosteo et al., 2015; Passarelli, 2015) and consequently they are likely to display a greater variety of goal-directed behaviors soon after goals are set. This program therefore makes it an ideal context for the development and validation of our scale.

### Step 1: Item Generation

A pool of 31 items was generated to assess GDB, ensuring that the items covered each of the four dimensions of the construct. We foresaw a final retention of four to six items per scale dimension and therefore proposed that approximately double the number of items be initially generated (Hinkin, 1998). Given that the scale is a self-report instrument, the items reflected the individual’s self-perception of the behaviors enacted to achieve the goals.

#### Sharing Information

To measure the first of the theoretically derived dimensions, a list of six items was generated (e.g., *I have explained my goals to…* and *I have shared my degree of plan implementation with…*). All the items were to be evaluated on a 5-point response scale ranging from 1 = only my coach to 5 = more than three people.

#### Seeking Information

To assess this second dimension, also deduced from theory, a list of an additional 11 items was created (e.g., *I have sought further information to help me better define my action plan* and *I have asked for advice regarding my feedback*). All the items were to be evaluated on a 5-point Likert scale (from 1 = strongly disagree to 5 = strongly agree).

#### Revising the Plan

Six items were created to measure this dimension, the only one inductively deduced from direct observations. Items such as *I have adapted my plan based on the information received* and *My plan after 3 months was different than my original plan* were added to the list, all to be evaluated on the same 5-point Likert scale.

#### Enacting the Plan

To complete the initial pull of items, a set of 12 items were generated to tap this last dimension of GDB (e.g., *I am putting the plans into practice*, and *I am progressing toward attaining my goals*). All items were also to be evaluated on the same 5-point Likert scale. We then screened the items to improve the wordings and eliminate redundancy. The number of items for the next development step was kept to 31.

### Step 2: Face and Content Validity: Initial Item Reduction

Face and content validity refers to the adequacy with which a measure assesses the construct of interest (DeVellis, 2012). First, practitioners corroborated that all items had adequate face validity, and next we followed the more structured and rigorous approach for testing content validity (DeVellis, 2012). To this end, the 31 items were analyzed and sorted following the proportion of substantive agreement (Anderson and Gerbing, 1991). Seven raters (four subject-matter experts and three PhD students from related research fields) were asked to sort the items into categories based on the dimension of the GDB construct that the items seemed to describe. The raters provided a description for each category and assessed how relevant each item was to its intended dimension (high, moderate, or low). Items that were consistently rated as highly relevant to the same dimension were kept. Items that were inconsistently classified as tapping different dimensions, and items whose relevance to the dimension was assessed as low or moderate, were reworded (as suggested by the subject-matter experts) or eliminated.

During this process of content validation, the inconsistent classification of the items that described discussing information led to a rewording of the items. The new wording made the intention of the goal-directed behavior clearer: the intention being either that of sharing information (to discuss just to share one’s intentions with others) or that of seeking information (to discuss in order to receive feedback). Some inconsistencies in the classification of some other items between the categories *Seeking Information* and *Revising the Plan* also led to additional rewording and item reduction. This process of content validation led to a preliminary GDB scale consisting of 25 items tapping the four domains of our GDB construct. The scaling was left as originally proposed.

### Step 3: Further Item Reduction (Study 1)

The purpose of Study 1 was to create a more parsimonious scale by further reducing the number of items based on the questionnaire’s psychometric properties, while maximizing internal consistency (reliabilities) among items (Hinkin, 1998). We also continued to explore the dimensional structure of the construct’s measurement instrument.

#### Sample 1

Study 1 targeted 355 international MBA students at a leading European business school, 157 of whom responded to the survey (44% response rate). The sample comprised 35 nationalities, the gender split was 64% men and 36% women, the mean age was 29.15 (SD = 3.06), and the mean work experience was 5.8 years (SD = 3.08). A sample size of 157 is sufficient to obtain an accurate solution in an exploratory factor analysis (EFA) if loadings are reasonably high (Guadagnoli and Velicer, 1988), which it was in our case^4^. The MBA students took part in an abridged version of the leadership development program (the full version available for the Executive MBA students included several coaching sessions that reinforced the vision-based process). Using a sample that is not from our target population is less critical at this exploratory stage of scale development. But nevertheless, the sample was similar enough as participants also worked on the vision and received 360° feedback as a base for their development plan.

#### Questionnaire Administration

A survey with the 25 items of the preliminary GDB scale was delivered *via* Qualtrics® software. The items were randomly mixed to diminish the threat of systematic measurement error due to similar items appearing sequentially in the survey. This randomization was done for all items except for the ones related to *Sharing Information*, as these had a different response scale that necessitated their appearing together. The questionnaire was preceded by the following instruction:

*Think of a time when you set some personal goals and defined the corresponding action plans, ideally at the end of a development or training program. For each item of this section, please assess the degree to which you showed the following behaviors during the first 3 months after setting your goals and plans*.

#### Data Analysis

Our initial assumption was that all items for each subscale were reflective. We therefore expected to find high inter-item correlations and all items to load onto one dimension for each subscale. Items within the same subscale with low inter-item correlations were plotted to check for outliers. A few outliers were detected, but they concerned only the response to one item (i.e., the individuals had clearly misunderstood the item and assessed it with an inconsistent answer). These values were recalculated using the SPSS EM maximum likelihood method (Cuesta and Fontseca, 2014).

To verify the underlying factor structure of the preliminary scale, we conducted for each subscale a factor analysis using maximum likelihood as the estimation criterion, and forcing the number of factors to one. We retained the items that loaded strongly onto the latent factor. We examined the nature of the items that did not meet these requirements to verify whether they were formative as opposed to reflective (i.e., tapping a new dimension within the subscale). Reflective items with poor loadings (less than 0.500) were either reworded or deleted. The elimination of three such items improved not only the parsimony of the scale but also its reliability, as the number of items was sufficiently high (Hinkin, 1998). We also verified that the total variance (of the items for each subscale) accounted for by the single factor exceeded the minimum 60% recommended value (Hinkin, 1998). Finally, realizing that the variability of the data was low, a shift from a 5- to a 7-point scale was adopted for all 22 remaining items of the GDB scale.

### Step 4: Second EFA and Final Goal-Directed Behaviors Scale (Study 2)

The purpose of Study 2 was to explore how to minimize the number of items while maintaining good psychometric properties of the scale.

#### Sample 2

For this second EFA, we targeted 185 new international MBA students at the same leading European business school. Ninety of them responded to the survey (48% response rate). The sample comprised 32 nationalities, the gender split was 75% men and 25% women, the mean age was 29.8 (SD = 2.60), and the mean work experience was 5.8 years (SD = 2.33).

##### Sharing Information

Loadings for the five items continued to be above 0.80, and the subscale showed an *α* coefficient of 0.94. The variance explained by one factor was 75.3%. Given these results, all five items were kept for the final GDB scale.

##### Seeking Information

The answers to one item (*I sought further clarification on the feedback I received*) lacked consistency with respect to the rest^5^. Without it, psychometric properties improved: variance explained by one factor increased to 51.9%, while the *α* coefficient stayed at 0.80 despite having one item less. In view of these results, the item was excluded from the final GDB scale.

##### Revising the Plan

Two items out of seven showed poor loadings onto the latent factor. Their wording revealed that the items were tapping a slightly different domain which was not considered especially relevant. Hence, to keep the scale unidimensional and parsimonious, both items were eliminated. A third item (*my plan after 3 months was different than my original plan*), although reflective, was also eliminated. We attributed its lower loading to the item’s specificity: the reference to a limited period of time that was unique among all five items. As a result, the scale for *Revising the Plan* was reduced to four items, the variance explained by one factor increased from 50 to 66% and the *α* coefficient remained high at 0.88.

##### Enacting the Plan

Loadings for all five items surpassed 0.71, variance explained by one factor was 62.1% and the subscale showed an *α* coefficient of 0.89. In view of these results, all five items were kept for the final GDB scale.

Results corroborated the reflective nature of all items and the unidimentionality of the subscales. An EFA (using maximum likelihood estimation criterion, promax rotation, and forcing the number of factors to four) provided more evidence for the four-factor model. All items but one loaded significantly higher on the latent factor that they were supposed to measure (with values above 0.73) than on the other factors of the scale. The exception was one item from *Sharing Information* that loaded slightly higher on *Enacting the Plan.* We did not attribute this cross loading to the latent factor but to the fact that the item shared a wording specificity with one item of *Enacting the Plan* (which we later confirmed in the CFA^6^). Consequently, the item was kept and a final 18-item, 4-dimensional GDB scale was proposed (Table 1).

**Table 1:** General scale of goal-directed behaviors (GDB).

Sub-scale		Item
Sharing information	1.	I shared my degree of plan implementation with…
	2.	I shared relevant information about my goals and plan with…
	3.	I explained my goals to…
	4.	I talked about my plan to reach my goals with…
	5.	I gave details of my plan to…
Seeking information	6.	I sought further information that is relevant for my plan
	7.	I sought feedback from others about my goal intentions
	8.	I asked for people's comments about my plan
	9.	I looked for feedback about the initial steps that I have taken
Revising the plan	10.	I modified the action plan to better achieve my goals
	11.	I redefined the strategy to attain my goals
	12.	I adapted my plan based on the information obtained
	13.	I modified the plan using the information that I acquired
Enacting the plan	14.	I took steps towards implementing my plan
	15.	I made decisions that were congruent with my goal intentions
	16.	Putting the actions into practice helped me advance towards my goals
	17.	I progressed towards my goals
	18.	I started to implement some of the actions in my plan

All items measured on a seven-point response scale. Sharing Intentions: 1 = nobody or only my coach/2 = one person/… /7 = more than five people. Rest of sub-scales: 1 = strongly disagree/2 = disagree / 3 = somewhat disagree /4 = neither agree nor disagree/5 = somewhat agree/6 = agree/7 = strongly agree.

### Step 5: Reliability and Average Variance Extracted (Study 3)

The purpose of Study 3 was to evaluate the GDB scale by reassessing its psychometric properties and establishing construct validity for each of the dimensions underlying the questionnaire.

#### Sample 3

This last study targeted students from four cohorts of the Executive MBA program from the same leading European business school as the previous samples. Executive MBA participants took the full version of the leadership development program which included several seminars and vision-based coaching sessions to assist participants in each phase of their personal change process. As previously stated, this was the ideal context for the final evaluation and validation of our scale. The study targeted 170 students, 86 of whom completed the survey (51% response rate). The gender split was 72% men and 28% women, the mean age was 35.2 (SD = 4.52), the mean work experience was 10.2 years (SD = 4.23), and 12 nationalities were represented (81% from Spain).

#### Extended Questionnaire

For construct validation purposes, the survey, administered through the Qualtrics platform, included the scales of the constructs from the nomological network of goal attainment, whose conceptual relationship with our GDB construct we hypothesized in the theoretical section of the paper. The survey also collected biographical data through close-ended questions.

#### Data Analysis

In a first exploratory stage, several outliers concerning the response of one item were detected and their values imputed. One individual appeared as a persistent outlier in most of the plots and was therefore excluded from the analysis, reducing the sample size to 85 individuals.

#### Reliability and Average Variance Extracted

All four subscales measuring GDB were found to be unidimensional and composed of reflective items. Internal consistency reliabilities were therefore assessed with Cronbach’s alpha, and with Heise and Bohrnstedt’s omega coefficients, the latter of which is recommended when items are not Tau-equivalent (Deng and Chan, 2017), as is clearly the case in *Seeking Information.* Average Variance Extracted (AVE; i.e., average communalities extracted per subscale) was also calculated. Results revealed good psychometric properties for all of the subscales (Table 2).

**Table 2:** AVE, Cronbach’s alpha, and omega of the 4 GDB sub-scales.

	AVE (%)	α	Ω
Sharing information	77.4	0.94	0.90
Seeking information	61.7	0.86	0.78
Revising the plan	64.1	0.87	0.80
Enacting the plan	55.1	0.86	0.78

### Step 6: Goodness-of-Fit (Study 3)

As presented earlier in the paper, our aggregate model of GDB (Figure 1) hypothesizes the relationships among the four dimensions that form the construct. Study 3 also sought to evaluate the goodness-of-fit of our measurement model.

First, a CFA was performed to verify the measurement quality of the factor structure, and to provide first evidence of construct validity of the new GDB scale (Jöreskog, 1969). All CFA loadings of the indicators related to each factor were well above 0.70 (>0.84 for Sharing Information, >0.72 for Seeking Information, >0.75 for Revising the Plan, and > 0.70 for Enacting the plan). Details are provided in Table 3.

**Table 3:** CFA measurement model. Loading estimates.

		Factor			
		1	2	3	4
Information sharing	Item 1	0.840			
	Item 2	0.897			
	Item 3	0.931			
	Item 4	0.959			
	Item 5	0.901			
Information seeking	Item 6		0.716		
	Item 7		0.787		
	Item 8		0.837		
	Item 9		0.828		
Revising the plan	Item 10			0.885	
	Item 11			0.824	
	Item 12			0.746	
	Item 13			0.785	
Enacting the plan	Item 14				0.693
	Item 15				0.712
	Item 16				0.872
	Item 17				0.838
	Item 18				0.802

Completely standardized solution.

Conclusions from CFA results cannot be drawn without assessing the goodness-of-fit of the model first. Despite not having a large sample size, the high loadings revealed by the CFA rendered enough power to the goodness-of-fit test (Saris et al., 2009), and thus allowed us to confidently interpret the test results.

**Figure 3:**
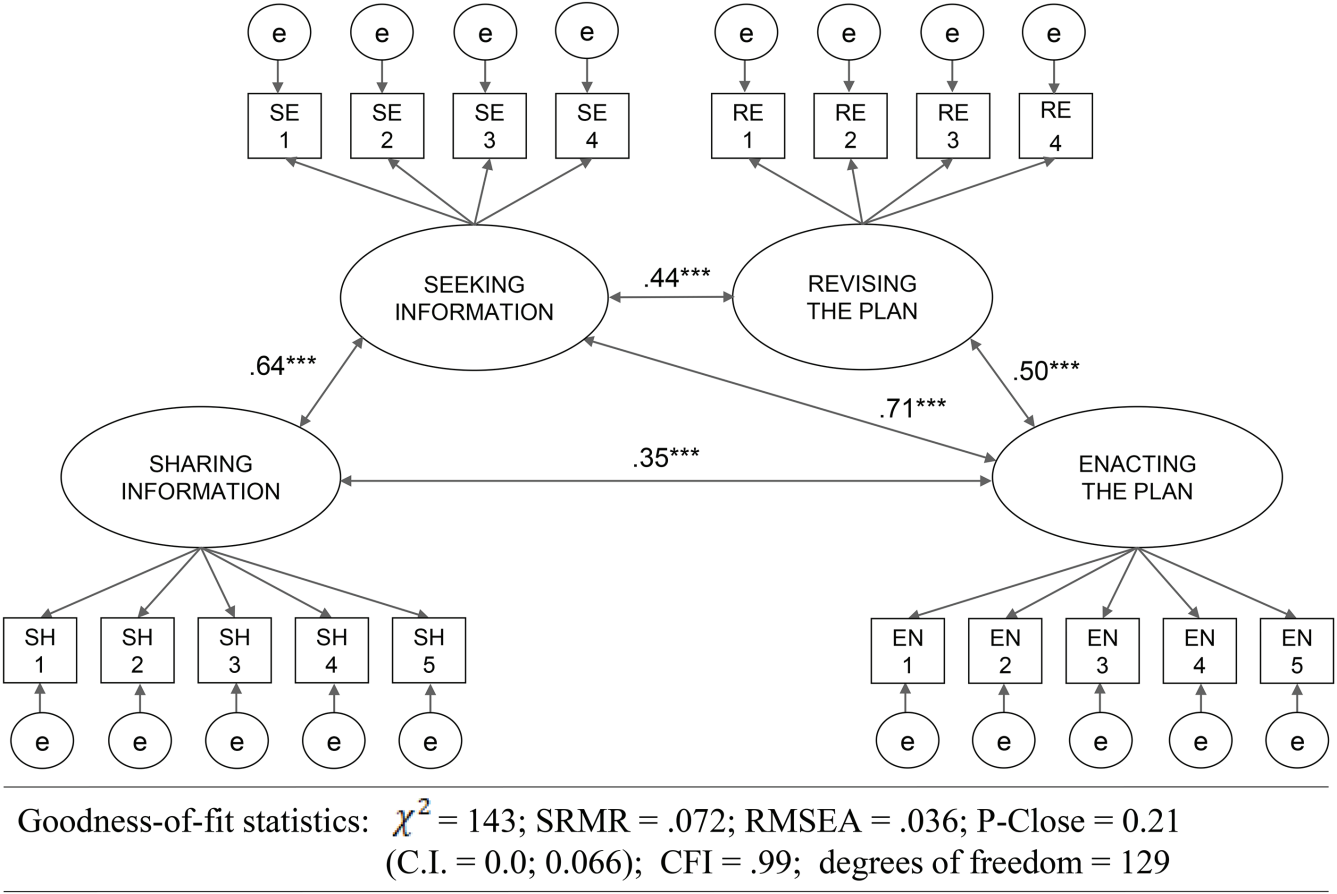
Model of GDB and CFA statistics. Correlations between the four dimensions of the scales and goodness of fit statistics.

CFA using the data from Sample 3 resulted in good global fit indices (Figure 3). All global indices, such as the χ^2^/df ratio, Root Mean Square Error of Approximation (RMSEA), Square Root Mean Residual (SRMR), and Comparative Fit Index (CFI) were above the usual thresholds (Hu and Bentler, 1999).

Bivariate correlations^7^ among the four dimensions were found to be highly significant (Table 4) for the five relationships hypothesized. Correlation between *Sharing Information* and *Revising the Plan* was nonsignificant, as predicted in our model. In conclusion, results from the CFA support the 4-factor structure of our model, and provide first evidence of construct validity by clearly discriminating the four dimensions within the GDB construct.

**Table 4:** Descriptive statistics and zero-order correlations for sample 3.

Variable	M	SD	1	2	3	4	5	6	7	8	9	10	11	12	13	14
1. Sharing information	3.66	1.80														
2. Seeking information	4.53	1.29	0.548***													
3. Revising the plan	4.54	1.35	0.200*	0.407***												
4. Enacting the plan	5.62	68	0.338***	0.591***	0.388***											
5. Goal commitment	4.08	72	0.319***	0.543***	0.205*	0.505***										
6. Learning goal orientation	4.54	0.39	0.288***	0.334***	0.268**	0.318***	0.309***									
7. Proving–PGO	3.37	67	0.015	0.100	0.144	0.024	0.122	0.014								
8. Avoiding–PGO	2.41	74	−0.119	−0.174	0.016	−0.281***	−0.151	−0.385***	0.253**							
9. Self-efficacy	4.10	45	0.169	0.224**	0.147	0.272**	0.144	0.448***	0.141	−0.209*						
10. Empathic concern	3.76	54	0.020	0.132	−0.023	−0.073	0.071	0.055	−0.035	−0.054	−0.125					
11. Career satisfaction	3.59	82	0.152	0.233**	0.020	0.211*	0.148	0.006	−006	−0.041	0.064	−0.115				
12. Gender	.27	45	−0.032	0.090	−0.070	−0.162	−0.019	0.040	0.194	0.121	−0.138	0.115	−0.004			
13. Age	35.20	4.52	−0.085	−0.071	0.181*	0.002	0.005	0.079	−0.145	0.109	−0.084	−0.053	0.021	−0.068		
14. Tenure	10.18	4.23	−0.087	−0.045	0.079	0.002	0.014	0.083	−0.180*	−0.144	−0.002	0.013	0.055	−0.013	0.793***	

N = 85. Gender was coded as 1 (female) and 0 (male).

PGO, Performance goal orientation.

* p < 0.10

** p < 0.05

*** p < 0.01.

### Step 7: Convergent and Discriminant Validity (Study 3)

To gather further evidence of construct validity, we assessed convergent and discriminant validity, by testing the conceptual relationships between the newly developed GDB scale and the six proposed measures from the nomological network of goal attainment: goal commitment, self-efficacy, learning goal orientation (LGO), proving performance goal orientation (PPGO), avoiding performance goal orientation (APGO), and empathic concern. Bivariate correlations between constructs are presented in Table 4.

#### Convergent Validity

As predicted, we found evidence of the positive association between some dimensions of GDB and Goal Commitment, LGO and Self-Efficacy, and of the negative association between some dimensions of GDB and Avoiding-PGO.

Bivariate correlations between Goal Commitment and GDB were positive and highly significant for three of the scale dimensions: *Sharing Information* (*r* = 0.32), *Seeking Information* (*r* = 0.54), and *Enacting the Plan* (*r* = 0.51). LGO was also positively correlated with *Sharing Information* (*r* = 0.23), *Seeking Information* (*r* = 0.33), and *Enacting the Plan* (*r* = 0.32). Also, as expected, General Self-Efficacy positively correlated with *Seeking Information* (*r* = 0.22) and *Enacting the Plan* (*r* = 0.27). Altogether, these results supported the convergent validity of our scale. Regarding Avoiding-PGO, bivariate correlations with our GDB dimensions were negative and significant for Enacting the Plan (*r* = −0.28), and negative but not significant for the other GDB dimensions. Construct validity, in this case, was partially supported.

#### Discriminant Validity

Discriminant validity of the GDB scale was assessed firstly by finding evidence of the lack of correlation between GDB and two constructs in the nomological network that we predicted to be unrelated to goal attainment, Proving-PGO and Empathic Concern. As expected, none of the bivariate correlations (Table 4) between either of the two constructs and the four dimensions of the GDB were significant, thus supporting the discriminant validity of our scale.

Additionally, the assessment using the Fornell-Larcker criterion also supported the discriminant validity of the GDB scale in relation with the related constructs of Goal Commitment, Self-efficacy, and LGO. In our case, the AVE value of each GDB subscale exceeded the squared correlations between the GDB subscale and the related constructs (more than double in all cases).

## General Discussion

Business schools, through their executive education programs, are increasingly attending their participants’ needs to embark on a personal or professional transition (Russon and Reinelt, 2004; Kets de Vries and Korotov, 2007). Although leadership development programs encourage participants to establish a personal development plan, schools seldom know if participants actually meet their goals and succeed in realizing the intentional change process. Goal attainment is considered a key indicator of the impact that leadership development programs have on their participants (Yammarino, 1993; Toegel and Conger, 2003), but its measurement constitutes a real challenge since goals vary greatly in nature, and years may elapse before goals are fully achieved.

Although we can find measures of goal attainment in the context of leadership development programs, such as a second 360-feedback, self-reported scales on specific competencies (e.g., Black and Earnest, 2009), and the Goal Attainment Scale (Spence, 2007), none overcome both challenges of being able to measure progress toward multiple goals of different nature, and being able to do it early enough to make data collection feasible.

In this study, we sought to overcome both challenges and contribute to the literature on leadership development with a general scale of GDB, which measures four distinct general behaviors that are instrumental to goal attainment, and that can be applied as early as a few months after goals are set. Those who succeed in achieving their goals are more likely to (1) share their goal and plan intentions with more people, (2) engage in the search for information or better strategies to achieve their goals, (3) improve or adapt the plan associated with the goals, based on the information obtained, and (4) start implementing the actions of the plan.

The application of the scale to our target population (85 professionals who participated in a leadership development program in executive education) evinced the advantages of this new measurement instrument. First, we were able to collect data 3 months after individuals had set their goals, a time that coincided with the end of the Executive MBA program and therefore led to a response rate as high as 51%. Second, the scale captures four general behaviors that manifest when individuals engage in their change process, regardless of the number or nature of the goals and action plans that participants establish. Therefore, measuring goal progress 3 months after goals are set seems to be early enough to facilitate data collection, but it is late enough for individuals to be less biased by the honeymoon effect of the training.

### Contribution

The development of our general scale of GDB has both theoretical and practical implications. First, we fill a gap in the leadership development literature (Hooijberg and Lane, 2009) by providing a proximal measure of goal attainment developed to assess the short-term impact of leadership development programs. Most specifically, the new scale is most indicated to assess programs designed around Intentional Change Theory (ICT) (Boyatzis, 2006, 2008) as it captures the degree of engagement in goal pursuit through some general goal-directed behaviors that such vision-based coaching programs seek to promote. Since coaches assist their clients with the definition of their personal vision, goals are more likely to be set in a context of a long-term aspiration, and the change process is more likely to induce the positive emotions required to sustain goal striving (Boyatzis, 2006, 2008; Howard, 2015; Passarelli, 2015). Such conversations with the coach leading to the articulation of a well-defined vision may facilitate similar conversations with people other than the coach and therefore promote *sharing information* with others, which is the first behavior captured by the scale. Additionally, positive emotions activate a psychophysiological state that makes individuals cognitively more open to exploring new ideas and experiences (Fredrickson, 2001; Boyatzis et al., 2015; Passarelli, 2015). Consequently, the ICT process is also likely to facilitate behaviors such as *seeking information* on how to better attain the goals, *revising the plans* if needed and eventually taking the first steps to experiment, (i.e., *enacting the plan*), behaviors that are also measured by the scale.

Second, the study also contributes to goal setting theory as all hypotheses regarding the relationships among scale dimensions were supported. Results add to the already mounting evidence of the benefits of making goal intentions public (Hollenbeck et al., 1989a,b; Epton et al., 2017), and the benefits of seeking information relevant to the goals (Locke and Latham, 1990, 2002; Latham, 2004; Seijits and Latham, 2005; Harkin et al., 2016). Both behaviors (and revising the plan) were all shown to be positively correlated with enacting the plan, and hence all likely to positively influence progress toward the goals.

The possibility of data collection as early as a few months after goals are set also has implications for practice. By means of our GDB scale, institutions (e.g., business schools and universities) will be able to easily measure the degree to which leadership development programs help their participants engage in their personal change process. With this information, institutions may be able to assess the impact of their programs by comparing the average GDB among cohorts and analyze if this average improves over time as a result of the program upgrades or interventions (such as improving the goal setting process or the coaching process). These institutions may also use this information to externally promote their leadership development programs among future potential participants.

Finally, executive coaches may put more emphasis on prompting their coaches to engage in each of the four GDB by, for example, the articulation of these behaviors in their action plan. Coaches could also be encouraged to reflect on their self-assessed GDB as a self-regulatory strategy, which is likely to motivate corrective actions that help with progress toward the goals.

### Limitations

This study has some limitations. First, the general scale of GDB is a self-reported scale, and therefore its assessment is susceptible to being biased by social desirability, which may pose a threat to construct validity. We consciously did not control for social desirability in the survey. Long questionnaires produce respondent fatigue and carelessness (Hinkin, 1995), and increase the likelihood that participants drop the survey before completion. For this reason, besides the items of the GDB scale, we chose to include only the most relevant constructs for testing convergent and discriminant validity. However, this threat was minimized by the fact that the answers to the study were not linked to any program results, and that the surveys were anonymous.

Second, the new scale operationalizes GDB by measuring the individual’s self-perception of the construct. This constitutes a threat to construct validity due to mono-operation (Shadish et al., 2002). External and more objective measures of GDB (e.g., ratings by others) would provide further evidence of convergent validity. In addition, using the same method (i.e., also self-reports) to operationalize the rest of the constructs could generate common-method bias. Despite these threats, empirical correlations (positive, negative, and no correlations) strongly matched the associations between constructs that the theory predicted.

Third, no test for criterion validity was performed. Since GDB is conceptualized as a proximal measure of goal attainment, a longitudinal study should ideally be conducted to test the extent to which the measure predicts goal attainment, thus assessing the predictive validity of the construct.

The relatively small sample (90 individuals in Sample 2, 85 in Sample 3) can be a threat to the statistical conclusions validity of the study. Sample size for EFA is recommended to be in an item-to-response ratio of at least 1:4 (Rummel, 1970), or around 150 as long as correlations among items within each dimension are sufficiently strong (Guadagnoli and Velicer, 1988), which turned out to be the case for our GDB scale. In addition, the high loadings (all above 0.70) rendered high power to the tests of goodness-of-fit (Guadagnoli and Velicer, 1988), and thus helped to diminish this threat.

Finally, this scale has been developed and validated in a context where goals are self-set and typically concern the development of leadership competencies or more general career or life aspirations. In such a context, behaviors such as sharing information, seeking information, and revising the plan appear to be highly relevant to goal attainment. The generalizability of the scale to contexts that do not fulfill such conditions is therefore questionable.

### Directions for Future Research

The general scale of GDB broadens the opportunities for research in goal-striving contexts where goals vary greatly among individuals. For example, as the new GDB scale is most appropriate for measuring the impact of leadership development programs in executive education, it may allow further validation of some of the central tenets of Intentional Change Theory through the use of our GDB scale to compare the impact of coaching to vision with that of coaching for improvement needs (see Howard, 2015).

Regarding research on the scale itself, future research should address the criterion validity by assessing, through longitudinal studies, the predictive power of GDB on measures of goal attainment (e.g., self-reported assessment or second multisource feedback). The scale of GDB has laid the first stone for building a predictive model of goal attainment (by including constructs that would further explain the variance in goal attainment). Further research should also aim at discovering possible underlying causal processes among the four dimensions, which would render explanatory power to the model (Sutton, 1998). As a first step, we suggest exploring the effect that goal commitment might have on the predictive and explanatory power of the model. We would expect that goal commitment is likely to at least partially explain the positive relationship between Sharing Information and the two dimensions of GDB with which it correlates. We would also expect some behaviors to occur in a certain temporal sequence, as *Revising the Plan* seems to function as a partial mediator between *Seeking Information* and *Enacting the Plan*. Further research should therefore seek to further understand a possible temporal sequence among the dimensions within the model, as this would be valuable information for guiding executives on the steps to follow.

Goals and action plans in leadership development programs led by business organizations (as opposed to business schools) are usually more straitjacketed: goals and action plans are typically work-related and shared by the boss or other managerial functions. Testing the scale of GDB in such contexts would contribute to the assessment of its external validity.

Future research should also examine how the structure of goals and action plans relates to each of the GDB. Findings from such research could have practical implications since they could serve as guidance for practitioners to improve the goal setting process. This could open the door to studies using quasi-experimental designs where an intervention (e.g., coaches encouraging participants to plan their intentions to enact each of the four GDB) could be applied to an experimental group, to then determine the significance and the size of the effect on GDB with regard to the control group.

In conclusion, the general scale of GDB generates opportunities for future research in the field of leadership development, research that should help academics and practitioners in their quest for making these leadership development programs more effective, and for better guiding their participants to fulfill their personal and professional aspirations.

## Ethics Statement

This study was carried out in accordance with the guidelines regarding the Use of Human Subjects in Research issued by the ESADE Research Ethics Committee, affiliated to the Ramon Llull University of Barcelona. The study has been reviewed and approved by the ESADE Research Ethics Committee. Written informed consent was obtained from all research participants. A copy of the Application Form for Ethical Review (Approval number 002/2017) is available at request to the principal author.

## Author Contributions

The three authors fullfill the criteria established by the International Committee of Medical Journal Editors in Frontiers. They have substantially contributed to the conception or design of the work, and the analysis and interpretation of the data; helped in drafting the work and revised it critically for important intellectual content; provided approval for publication of the content; agreed to be accountable for all aspects of the work in ensuring that questions related to the accuracy or integrity of any part of the work are appropriately investigated and resolved.

### Conflict of Interest Statement

The authors declare that the research was conducted in the absence of any commercial or financial relationships that could be construed as a potential conflict of interest.

## Footnotes

^1^The term leadership development is used throughout the article as a generic term for leader and leadership development.

^2^A specific code was developed to assess goal nature. Goals were assessed by two expert coders, yielding an interrater reliability of 88%.

^3^The literature review comprised 86 articles (25 conceptual and 61 empirical), published in Management and Psychology journals with impact factor > 1, and covering 25 years of academic research.

^4^Only one item had a low loading, and thus was a candidate for elimination. Four items had loadings above 0.40, and all the rest had items well above 0.60.

^5^The fact that the leadership program already offers a coaching session to clarify feedback may lead to diverse interpretations of this item.

^6^The EFA had mistakenly shown a cross-loading because such an exploratory approach does not allow measurement errors from different items to correlate. CFA results confirmed that it was due to item 6 and item 14 (Table 1) sharing a specific wording. Allowing their respective specificities to correlate, this cross-loading no longer appears with the data from Sample 3.

^7^All input data not reported in the article are available on request from the first author of the paper.
